# The Hygiene of the Hair

**Published:** 1887-05

**Authors:** George Thomas Jackson


### Selections.

### *THE HYGIENE OF THE HAIR.*

By GEORGE THOMAS JACKSON, M. D.

Attention to the care of the hair and the hairy scalp is of special importance to those who belong to families in which premature baldness is hereditary; and it cannot be given too early. We should, therefore, instruct the parents as to the importance of giving attention to their children's heads, so that the matter may not be delayed too long, and the hair fall out when it is too late to stop it. Dandruff is regarded by most people as merely an annoyance, and, if not excessive, is neglected. If we could convince the laity that dandruff is one of the chief causes of baldness, it would eagerly seek relief, the disease could be early checked, and the day of hair-fall very much delayed. The care of the hair is important not only to those with an inherited tendency to baldness, but to all who wish to preserve their hair in good condition. If properly attended to, it will be a prophylactic not only to diseases of the hair proper, but also to parasitic troubles of all sorts. It is true that this demands the expenditure of a certain amount of time, but it is time well expended, though, I must confess, often greatly begrudged by male patients.

The hygiene of the hair and scalp consists in the proper use of the shampoo; in brushing and combing; in arranging the hair, especially in women; in the exposure of the hair to air and light, especially in men; in cutting and shaving it; and in the use, or rather avoidance, of pomades. We should watch over the hair from infancy, and instruct our patients as carefully in regard to its hygiene as we should do in regard to matters of general hygiene.

### SHAMPOO.

The first attention that the hair demands is the ridding of the scalp of the new-born child of the *vernix caseosa*. This is the first shampoo and should be more carefully performed than any subsequent one. Improper management at this time may entail endless worry to the mother and a great deal of suffering to the child, as it is exceedingly likely to set up an inflammation of the scalp.

The child is born covered with a fatty matter called *vernix caseosa*, which is often very thick upon the scalp. Steps are to be taken at once for its removal, which must be effected with the greatest care and with the avoidance of all force. To this end the scalp is to be saturated with sweet-almond oil, which is the most elegant means, or with olive oil or vaseline. It is preferable to use these in their natural state, but if desired there is no objection to perfuming them with a few drops of the oil of bergamot, wintergreen, or the like. The nurse should do this immediately after she has washed the child's face and eyes. Then, after the body has been bathed and the infant dressed, the head should be washed with plenty of warm water and soap, such as pure castile, or glycerin soap, either solid or liquid. This should be done very gently, and if the *vernix caseosa* is not readily removed, re-apply the oil and wait until the next day, when it will be easily washed off. Should it still prove obstinate, patiently repeat the process until it comes off. In no case use the fine toothed comb. For some weeks, the infant's scalp should be lightly oiled, as this will prevent any accumulation of sebaceous matter, and protect the tender skin from injury from atmospheric causes until the hair grows, care being taken to wash the head daily to prevent the oil from becoming rancid. When the hair is grown, the scalp need not be so often oiled, nor should it be washed more than twice a week.

In children and adults, the scalp should be kept clean, so as to avoid stopping up the hair follicles with foreign matter and to prevent any irritation of the scalp which its presence might cause. This is accomplished by the systematic use of the shampoo, followed by careful drying and the application of some oily substance to the scalp. It may be given as a rule that a shampoo every second to fourth week is sufficient for the scalp of those who are not exposed to more than the usual amount of dust; while those who are so exposed should shampoo their heads every week or two.

The daily practice of sousing the head with cold water, which is very commonly done by men, is pernicious, not because the water itself is harmful, but because the scalp is not properly dried afterwards; no oil is applied to take the place of the oil that has been removed by the water; the wet hair cannot bethoroughly brushed, and soon gets into a condition of dryness and brittleness. Women avoid getting their hair wet, and this may be one reason why they are less often bald.

The proper manner of shampooing the head is as follows: Choose some good soap, such as "Pear's Glycerine Soap," "Sarg's Liquid Glycerine Soap," pure castile soap; the tincture of green soap, or the tincture of prepared olive soap, and with plenty of warm water make a good lather on the head, and rub the head vigorously with the fingers, or with a rather stiff, long bristled brush. If the scalp is very sensitive to irritants, borax and water may be used instead of soap, or a mixture composed of the yolks of three eggs beaten up in a pint of lime water, either of which will make a good lather with water. When the head has been thoroughly shampooed, wash out the lather with a copious supply of warm water, or, where practicable, with alternate douches of warm and cold water, and then dry both scalp and hair with a good bath towel. When all is dry, rub on the scalp, not on the hair, a small quantity of some unctuous substance, such as sweet-almond oil, or vaseline. Care must be used in drying the hair, specially in women, who should sit before an open fire or in the sunlight in so doing; and who should not dress the hair until it is perfectly dry. To oil the scalp, the hair should be parted and the oil rubbed in along the part, then another part made and the operation repeated, and so on till the whole scalp is gone over. Should there be an excess of oil upon the hair, a condition which is disagreeable to many, it may be removed readily by pulling the hair between the folds of a damp towel moistened with ether, chloroform, or cologne water.

### BRUSHES AND BRUSHING.

Of far more importance than shampooing is the use of the brush and comb, and much more care should be given to the selection and use of these common toilet articles than is usually bestowed. Too often they are badly made, and generally, especially with men, they are used in a very perfunctionary manner. The brush which is to be used upon an infant's head should have long soft bristles, so as not to scratch or irritate the tender scalp, and should be employed simply in smoothing and polishing the hair. For young children whose hair is well grown, a stiff brush is necessary, and for adults two brushes should be used, a stiff one and a soft one. A properly-made brush has its bristles placed in little clumps or groups in such a manner that the middle bristles of each group are longer than those of the periphery. The bristles are well set into the back of the brush, and the groups are wide apart. Most of the brushes met with in the shops are made with the bristles all of the same length and the groups close together, so as to look pretty, but not to perform their proper function. The brush should be used systematically in the morning, and with considerable vigor, so as to produce a feeling of warmth in the scalp, and to brush out all particles of dandruff and foreign matter lodged in the hair. Every part of the scalp should be gone over with a stiff brush, and then it should be laid aside for the rest of the day, and the soft one used to assist the comb in parting the hair, and to give smoothness and gloss to it. The stiffness of the brush and the vigor of its employment must vary with the tenderness of the scalp, and in no case should be sufficient to cause a feeling of soreness. Were brushing performed in the manner indicated, the hair would lie properly without the aid of water or pomades, excepting, of course, in cases of mal-position of the hair, as in the so-called cow-lick, or where the hair is unnaturally stiff.

### COMBS AND COMBING.

The comb is next in importance to the brush, its office being to open up the hair so that the brush may reach all parts of the scalp, to part the hair, and to disentangle snarls. A properly-made comb has long, thick, wide, perfectly smooth teeth with well-rounded ends, and set wide apart. In choosing a comb, it should be held up to the light and discarded if any roughness or irregularities are found in the surfaces of its teeth, for such a comb would catch and tear the hair. Combs are usually made with a coarse and a fine half, and there is no objection to this arrangement if the fine part is used only to disentangle the hair. No attempt should be made to pick off crusts from the scalp with the comb. It should be used only as an assistant to the brush, and always with it in the systematic morning brushing. No comb should touch an infant's scalp, and the fine-toothed comb should be rigorously excluded from the toilet case. It is a dangerous instrument, the cause of many a case of eczema, and only of use in removing the ova of lice from the hair. Above all things, the tender scalp of the infant should be spared from its damaging effects.

### DRESSING OF THE HAIR OF WOMEN.

Now we come to a place in the discussion of the hygiene of the hair, in which fashion often interferes. Examination of old fashion-plates and portraits will show how women and men have tortured the hair, twisting it into all sorts of shapes and smothering it under wigs, false hair, and powder. Happily, at present the hair is worn more simply, but still the crimping or the curling iron is too much used, and the hair is pulled and dragged upon too much in adapting it to the varying demands of the hair-dresser. Sooner or later nature is apt to rebel against fashion and the hair of the woman is lost in the fight.

The simplest mode of wearing the hair is the best. It should be combed and brushed smoothly back upon the top of the head, either parted or not, as is most becoming, and gathered into a loose braid or coil at the back of the head. Girls should wear a pendant braid, and women whose hair is grown and who gather the hair into a coil, should use large hair-pins in fastening it, preferably of rubber or bone, with absolutely smooth surfaces. In doing up the hair, care should be taken not to drag upon it; and drawing it into unnatural positions, such as pulling the hair from the back of the head over forwards to near the forehead, should be avoided. If a woman's hair curls naturally, she should be thankful for the favor therein bestowed, but should it not curl of itself, she should not attempt to make it curl by singeing and squeezing it between hot irons, scorching it over a hot pipe-stem, or twisting it up tightly in curl papers.

WIGS, HATS, ETC.

The hair requires for its growth and for the maintenance of its health both air and sunlight, though not necessarily exposure to the direct rays of the sun. It is difficult to prove that wearing of the hat constantly is a cause of baldness, but there are many indications that such is the case, and it is well to avoid keeping the head covered with an unventilated hat. If the occupation compels one to be out of doors most of the time, or exposed to draughts so that a hat or cap must be worn, it should be well ventilated so that the heat from the head may not become confined and the head more or less sweated.

The wearing of wigs and false hair is bad for whatever hair remains, and should not be practiced. The absurd "water-falls" of a few years ago, and the no less ridiculous powdered wigs of old times, are happily things of the past, and should never be revived. If a woman's hair is short and scanty, it is better to wear it cut short, and endeavor to stimulate its growth by attention to the scalp, than by wearing false braids to assume a beauty which she has not. Wigs heat the head and sweat the hair; false hair by its weight drags upon the feeble hair it is desired to fortify.

The wearing of night-caps was once a custom founded upon the need of keeping the head warm in the inadequately heated bed-rooms of our ancestors. With the improvement in house building and heating, the custom has passed away, and should not be revived, as it excludes the air from the hair continuously for a good part of the day. Of course, where there is no hair, and the bald individual is sensitive to the cold, there can be no objection to keeping the head covered with a wig by day and a cap by night.

HAIR CUTTING.

All men wear the hair short and employ a barber at varying intervals, according to their fancy. As far as the health of the hair is concerned, it is immaterial whether it is cut at longer or shorter intervals, but it is essential that it should be well cut, and a good barber is desirable. It should never be "shingled," as the barbers term an operation which consists in cutting the hair by a to and fro motion of the shears, as this tears and roughens the hair.

The hair of children, whether they be boys or girls, should be kept short until the seventh or eighth year of age, as the growing hair is a drain upon the nutrition of the body, and at this time of life all the nutritive forces should be expended in the growth of muscle and bone. The hair of a girl, after she has reached her eighth year, should be allowed to grow, as the less the hair is cut the softer it is, and for a woman a head of fine hair is much to be desired. But should the girl be so situated that her scalp and hair cannot be properly cared for, then she will have a better chance for a good head of hair later in life if it is cut when she is young.

The hair of women is seldom worn short, although of late some women have seen fit to adopt the style of wearing the hair like a man, along with his coat and waist-coat. It is quite common for the long hair of women to be split at the point. This should be looked for, and if found, the hair should be cut above the cleft. All ragged ends should be lopped off, and all weak hairs should be cut off near the head to improve their strength.

### SHAVING.

The shaving of the beard is regulated largely by fashion. Physiologically, it is best not to shave, for if we do we rob ourselves of a useful protection to the throat and lungs. As shaving often makes the hair grow coarser, it is often resorted to very early by the youth for the purpose of rendering the down of the lip or cheek more apparent. It would be better to endure the down for a time, as he would be rewarded by the growth of an elegant short beard. If one must shave, he should do it himself, and see that his razors are kept sharp. He should shave himself so as to avoid the risk of an infection with ringworm of the beard; if his razors are dull, he is apt to set up an inflammation of the hair follicles or skin. For shaving, a mild soap that forms a thick lather should be used, and after the operation, especially in cold or windy weather, the face should be powdered with simple rice flour, or fine corn starch.

### POMADES.

Punch's advice to a man about to marry is equally applicable to the use of pomades. It was: "Don't." Their regular use upon the healthy scalp is uncalled for. They are dirty, soon become rancid, and emit a foul odor, unless this is covered by some strong perfume, and they soil whatever the wearer's head comes in contact with. If the rules already given are followed, the hair will be smooth and have sufficient lustre for beauty without pomades. If the scalp is diseased, advice should be sought, and the proper remedies obtained.

Most of the greases advertised for the cure or prevention of baldness or grayness are useless, and some harmful. The powers of some have been vaunted upon grounds that are rather funny, as for instance bear's grease, because the bear is well covered with hair. "Bandoline" and the like sticky substances, as well as hair dyes, should not be used, as the former is bad for the hair, and the latter are not infrequently followed by loss of health from the poisons they contain.

In some cases, the hair becomes matted together in a tangled mass, especially that of women during prolonged illness. From whatever cause arising, care and patience will usually enable the mass to be unraveled, and the hair saved. To do this, it must be attacked, a little at a time, with oil, soap and water, and the fingers, and picked apart and combed straight. By proper care, the condition is avoidable in most cases. It would be very exceptional when a patient could not bear the combing of the hair with a coarse comb once a day, followed by plaiting it into one or two plaits. When this is done gently and quietly, it will prove refreshing, and will prevent any trouble with the hair during convalescence. If it cannot be done, then it is best to cut off the hair to one-half or one-third of its length, so that it will be less liable to tangle.

As the hair sympathizes with the general health of the body, the latter should be maintained in good condition by a wise conformity to the laws of health. By the proper combination of the hygiene of the body with that of the hair, it is possible for even one who is predisposed to premature baldness to ward off the evil day for years, and one who comes of a strong-haired family should, as a rule, not become bald or have any essential disease of the hair.—*American Lancet.*

